# The prevalence and economic burden of treatment-resistant depression in Thailand

**DOI:** 10.1186/s12889-023-16477-y

**Published:** 2023-08-12

**Authors:** Jirada Prasartpornsirichoke, Nuttorn Pityaratstian, Chayanit Poolvoralaks, Naphat Sirinimnualkul, Tanaporn Ormtavesub, Nimmavadee Hiranwattana, Sasitorn Phonsit, Teerayuth Rungnirundorn

**Affiliations:** 1 Department of Psychiatry, Faculty of Medicine, Chulalongkorn University, 1873 Rama IV Road, Pathumwan, Bangkok, 10330 Thailand; 2 Department of Psychiatry, King Chulalongkorn Memorial Hospital, Bangkok, Thailand; 3 Faculty of Psychology, University College London, London, UK; 4 Excellence Center for Sleep Disorders, King Chulalongkorn Memorial Hospital, Thai Red Cross Society, Bangkok, Thailand; 5 Faculty of Medicine, Chulalongkorn University, Bangkok, Thailand; 6 Department of Psychology, Faculty of Social Sciences, Kasetsart University, Bangkok, Thailand

**Keywords:** Major Depressive Disorder (MDD), Economic costs of an illness, Treatment-resistant depression (TRD), Direct medical costs, Intangible costs, Productivity loss, Fail-to-treat, Antidepressants, Unpaid caregivers

## Abstract

**Background:**

The objectives of this study were to investigate the proportion of treatment-resistant depression (TRD) among patients with diagnosed major depressive disorder (MDD) and undergoing antidepressant treatment, to estimate the economic cost of MDD, TRD, and non-treatment-resistant depression (non-TRD), and to examine the differences between TRD and non-TRD MDD in a Thai public tertiary hospital.

**Methods:**

This was a combined study between retrospective review of medical records and a cross-sectional survey. The sample size was 500 dyads of antidepressant-treated MDD patients and their unpaid caregivers. MDD patients’ medical records, the concept of healthcare resource utilization, the Work Productivity and Activity Impairment Questionnaire: depression and mood & mental state versions (WPAI: D, MM), the Class Impairment Questionnaire (CIQ), and the Family Experiences Interview Schedule (FEIS) were applied as the tools of the study. Pearson Chi’s square, Fisher’s Exact test, and independent T-test were employed for statistical analysis.

**Results:**

The proportion of TRD was 19.6% among antidepressant-treated MDD patients in a Thai tertiary public hospital. The results of the study indicated that several factors showed a statistically significant association with TRD criteria. These factors included younger age of MDD patients, a younger age of onset of MDD, lower body mass index (BMI), a history of suicide attempts and self-harm, as well as frequent smoking behavior. The annualized economic cost of TRD was 276,059.97 baht per person ($7,668.33), which was significantly higher than that of cost of non-TRD (173,487.04 baht or $4,819.08). The aggregated economic costs of MDD were 96.8 million baht annually ($2.69 M) if calculated from 500 MDD patients and unpaid caregivers. This contributed to the economic cost of TRD 27.05 million baht (98 respondents) and the economic cost of non-TRD 69.74 million baht (402 respondents).

**Conclusions:**

The economic burden associated with TRD was significantly higher compared to non-TRD among antidepressant-treated MDD patients. Specifically, both direct medical costs and indirect costs were notably elevated in the TRD group.

## Introduction

Major depressive disorder (MDD) is a prevalent psychiatric disorder that causes a persistent feeling of sadness and impairs the daily function of the affected. It is considered one of the most debilitating forms of mental illness and ranks as a leading cause of disability globally. In fact, the burden imposed by depression is comparable to or even surpasses that of other chronic conditions, including diabetes, which highlights its profound impact on individuals and society [1]. Epidemiological surveys estimated its lifetime prevalence at 16.6% [2]. The World Mental Health (WMH) survey in 2011 showed a 12-month prevalence of MDD ranging from 2.2% in Japan to 10.4% in Brazil [3]. There was a substantial difference in estimates, possibly due to methodological processes employed. The prevalence was also found to be almost identical in ten high-income countries at 5.5% and eight low- and middle-income countries at 5.9%, indicating the fact that depression is not just a simple “modern world health crisis” that affects groups of people of socioeconomic status. The median age of onset of depression, basic sociodemographic and environmental correlates, and symptom profile and severity of depression were found to be generally comparable across different countries and cultures [4, 5]. Discrepancies found between countries are almost entirely accountable for the availability of resources and treatments. That is, in high-income countries, 50–60% of severe MDD cases received appropriate treatment [6, 7]. On the contrary, in low-income countries, less than 10% of severe MDD cases receive proper treatment [6].

Treatment-resistant depression (TRD) is characterized by resistance to antidepressant medications, although there is no universal definition of TRD, and it remains controversial what exactly qualifies as TRD in terms of the number of antidepressant classes used in treatment, duration of pharmacotherapy, and number of unsatisfactory responses to medications [8]. However, in essence, TRD is generally defined as ‘failure to respond’ to two or more treatments in an ‘adequate dose and duration’ of antidepressants [9–11]. In this article, we use the terminology of TRD from previous studies which state that; TRD is considered an account when a MDD patient meets the criteria for a major depressive episode (MDE) and has inadequate responses twice after receiving two lines of antidepressants of adequate dosage for a sufficient duration [10–12]. According to previous international research, the prevalence of TRD in the MDD population ranged from 12 to 55% for reasons concerning empirical data and measurements. The varying estimates of TRD prevalence are accountable to the nonuniformity of the definition in the criteria and factors that constitute TRD, as well as methodological differences [13, 14]. Another reason is the diverse social environment and the unequal accessibility of the mental healthcare system [6, 7].

TRD has a profound impact not only on individuals but also on society as a whole [15]. It poses considerable therapeutic challenges for depression treatment and dramatically reduces the prognosis of patients and their quality of life [10]. TRD was associated with melancholic characteristics, a higher risk of self-harm and suicide attempts, a diminished response to treatment for comorbid anxiety disorders [16], and a higher probability of having a comorbid autoimmune and cerebrovascular diseases [17]. Previous research has suggested a potential association between TRD and an increased risk of dementia and Alzheimer's disease. Individuals with difficult-to-treat MDD have significantly higher risk ratios for developing dementia and Alzheimer's disease compared to those with easy-to-treat MDD [18]. Furthermore, a systematic review investigating the negative impact of TRD on health-related quality of life (HRQoL) found that on a scale of 0–1, self-rated scores were 0.26–0.41 points lower for the adult population with TRD relative to MDD in remission or those who responded to treatment [19]. In comparison to patients with MDD who did not meet the criteria for TRD or non-TRD, those with TRD experienced significantly longer durations of depressive episodes. The duration of suffering from depressive episodes in individuals with TRD was found to be at least twice as long as that of patients with non-TRD.

A previous study conducted in Latin American countries, including Mexico, Colombia, Brazil, and Argentina, revealed that the median duration of MDD among individuals with TRD was 8 years. In contrast, the non-TRD patients had a considerably shorter median duration of MDD, which was reported to be only 1.9 years [20]. The prolonged duration of MDD treatments caused physical, mental, and financial problems for both the patient and the society, most notably healthcare resources utilization, at individual, societal, and national levels [21].

The concept of the economic burden of TRD was employed to evaluate both the direct and indirect impacts of TRD. This evaluation encompassed tangible costs, such as healthcare expenditures and medication costs, and expenses associated with commuting to healthcare facilities, as well as intangible costs, including productivity loss and work absenteeism. Previous literature found that the economic burden of TRD was significantly higher than that of non-TRD [11, 22–25]. The annual incremental burden of TRD in the United States was approximately $43.8 billion. Notably, nearly half of the total economic burden associated with medication-treated MDD (47.2%) could be attributed to almost one-third of patients who met the criteria for TRD (30.9%). Furthermore, the remaining half of the total economic burden associated with medication-treated MDD was attributed to more than two-thirds of patients who did not meet the criteria for TRD [26].

Additionally, the estimated indirect costs of TRD were much higher compared to the direct costs of TRD, especially in the issue of job loss from TRD [27, 28]. As of now, the exploration of the economic burden of TRD in Thailand remains an unexplored area of research. Conducting a study to assess the economic burden of TRD in Thailand would contribute to filling this knowledge gap and provide important insights for policymakers, healthcare providers, and stakeholders in addressing the challenges posed by TRD and allocating resources effectively.

The primary objective of this study was to assess and estimate the economic burden specifically associated with TRD in Thailand. In addition to estimating the economic burden, the study also sought to explore the proportion of TRD among patients diagnosed with Major Depressive Disorder (MDD) and examine the differences between patients with TRD and those with non-TRD among individuals diagnosed with MDD at the patient-level analysis. By examining these objectives, the study aimed to provide a comprehensive understanding of the prevalence, clinical characteristics, and economic implications of TRD in the context of Thailand.

## Materials and methods

### Procedure

This study employed a combination of a cross-sectional survey and a retrospective review. Two research tools were utilized to gather data. The first tool involved the examination of medical records in a retrospective review, while the second research tool consisted of self-rated questionnaires, which were administered during the cross-sectional survey. In this study, an electronic case record form (eCRF) was utilized to extract various data elements from the medical records of MDD patients. This included information on the historical treatment received for MDD, utilization of medical activities, and specific details regarding the strength, dose, and frequency of antidepressants and other medications prescribed to the patients. Additionally, the data also captured the patients' response to previous antidepressant treatment when transitioning to a new antidepressant, considering factors such as mood stabilization and any minimal improvement in mood or life functioning observed. These data elements were obtained by a psychiatrist and focused on the period of the prior 18 months leading up to the data collection period.

Subsequently, a cross-sectional survey was used to gather information on participant’s sociodemographic characteristics, medical comorbidities of MDD, productivity loss from MDD, and unpaid caregiver information. All potentially eligible MDD patients who visited the psychiatric clinic at King Chulalongkorn Memorial Hospital (KCMH) for routine follow-up visits from 14 March to 19 July 2022 were invited to participate in this study. The KCMH is a public general and tertiary referral hospital located in Bangkok, the capital city of Thailand, with more than a thousand beds accessible for inpatient care. All participants were granted informed consent to participate in the study and gave permission to access their medical history. After MDD patients completed the questionnaires, if available, an eligible family member or unpaid caregiver who accompanied a participant on the day was invited to participate in the caregiver survey. MDD patients were asked to contact and inform their caregivers to participate in the research project by filling out an electronic questionnaire via Google forms if they visited the hospital alone. This study was carried out in full accordance with the Declaration of Helsinki and has been approved by the Institutional Review Board (IRB) of Chulalongkorn University, Faculty of Medicine (IRB: 999/64).

### Participants

**Fig. 1:**
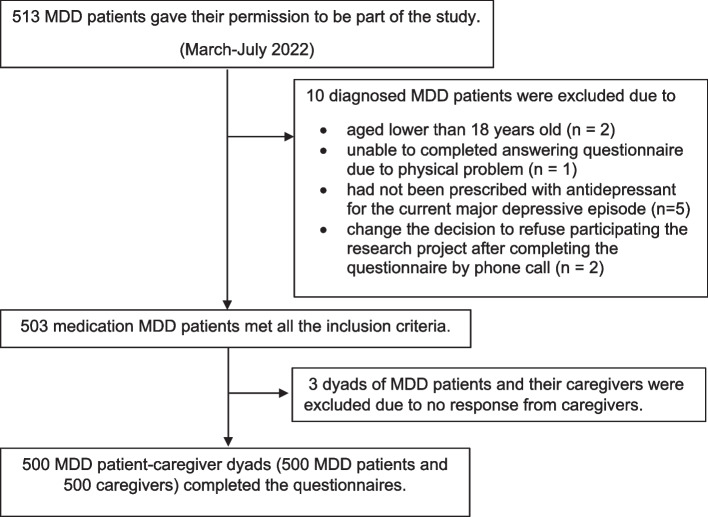
The Flow Chart of MDD Patients and Their Caregivers Included in the Study

The sample included 500 eligible MDD patient-unpaid caregiver dyads. MDD patients were included if they were 18 years old or older at the time of eligibility assessment and have a documented diagnosis of MDD (ICD-10: F32-F33, DSM-5: 296.2–296.3) based on clinical assessment by a psychiatrist for at least four months. Furthermore, eligible patients had to be prescribed antidepressants for the current major depressive episode within the past 18 months. MDD patients who were documented to have co-occurring neurocognitive disorders (ICD-10: F00.X, F01.X, F02.X, F03.X), psychotic disorders (ICD-10: F20.X, F21.X, F22.X, F23.X, F25.X, F28.X), or manic episodes (ICD-10: F30.X, F31.X) over the last 18 months were excluded from the study. The patient’s caregiver was recruited if he/she was family or an informal (unpaid) caregiver of the patient; aged 18 years or older on the day of the survey; and provided at least one hour of care to the patient in the past week (including texting and calling). The flowchart of MDD patients and their caregivers in this study is presented in Fig. 1.

### Measurements

#### Diagnosis of major depressive disorder (MDD)

In this study, the diagnosis of MDD was based on the clinical judgment of psychiatrists and was consistent with the medical record of the patients that documented ICD-10 (code F32.X-F33.X) and DSM-5 (code 296.2X-296.3X). The present study specifically targeted MDD patients who were undergoing antidepressant treatment. These patients were categorized into two distinct groups: those who met the criteria for Treatment-Resistant Depression (TRD) and those who did not meet the criteria for TRD, referred to as non-TRD.

#### Treatment-resistant depression (TRD)

We follow the definition and criteria for TRD from C.H., et al. (2019). Patients with MDD who had a treatment history of the use of two or more antidepressants with a minimum use of four weeks each at the daily adequate dose and did not respond to treatment twice were considered TRD. The failure of a treatment course was defined as an inadequate response to antidepressant treatment for MDD. This inadequate response was assessed on criteria, such as minimal or no improvement in mood or life functioning. These criteria were documented in the medical charts, providing objective evidence of inadequate response to treatment. All medications must be prescribed for at least four weeks before being considered for use as an antidepressant. Data related to prescriptions (medication name, strength, dose, frequency, and quantity dispensed) were retrieved from the medical chart view to determine TRD and non-TRD in MDD patients. The degree of resistance to treatment refers to the number of antidepressants used during the observation period of their MDD treatment.

#### Non-treatment-resistant depression (non-TRD)

In the present study, the term "non-treatment-resistant depression" (non-TRD) refers to patients diagnosed with MDD by psychiatrists and receiving antidepressant medication treatment. However, these patients do not meet the specific criteria for TRD as defined in this study. The assessment of non-TRD status was based on reviewing medical records from the preceding 18 months prior to data collection. It is important to note that patients classified as non-TRD in this study may potentially develop into TRD patients in the future if they do not adequately respond to two different types of antidepressant treatments.

#### Economic costs of MDD, TRD, and non-TRD

In this study, the estimated total economic burden of MDD, TRD, and non-TRD was aggregated from three main components: direct medical costs, direct non-medical costs, and indirect costs. The economic burden of MDD, TRD, and non-TRD was generated in annual terms. In this study, both the Thai baht, the national currency of Thailand, and the US dollar were utilized as cost units for financial calculations. The exchange rate used for converting between these currencies was set at 1 USD = 36 Baht, as provided by the Bank of Thailand (BOT) in the fourth quarter of 2022. In principle, the annual total cost of MDD, TRD, and non-TRD for the country would be calculated based on the average cost per case multiplied by the total number of cases per year. This value represented the economic burden of MDD, TRD, and non-TRD for society and the country.

#### Direct medical costs

The direct medical cost is the tangible cost related to hospital activities. In this study, the concept of healthcare resource utilization (HCRU) was applied to reflect the direct medical cost including outpatient visits, inpatient hospitalizations, emergency visits, medications, laboratory tests, psychotherapy, and occupational therapy. We estimated the direct medical costs of MDD and TRD from data from medical records using an electronic case report form (eCRF) as a research tool. Direct medical costs in the study were assessed using two different approaches. The first approach considered direct medical costs from the perspective of MDD patients who utilized health insurance coverage. This approach involved considering the implementation of public and private health insurance to represent the direct medical costs that MDD patients pay for their MDD treatment. Typically, health insurance fully covers the overall direct medical costs. However, it is important to note that this method may not accurately reflect the actual medical costs associated with MDD treatment due to potential distortions caused by insurance coverage. The second approach involved calculating the direct medical costs without considering health insurance implementation. This approach considered the number of medical services provided to a patient and the corresponding billing charges before deducting any health insurance coverage. By considering the costs incurred by MDD patients in the healthcare system (the societal view including the healthcare provider’s costs), this method aimed to estimate the actual costs borne by the healthcare provider in delivering specific services [29]. This approach provided a more accurate representation of the true costs of medical services without the influence of insurance coverage. By employing these two different approaches, the study aimed to capture a more comprehensive understanding of the direct medical costs associated with MDD treatment, accounting for both the perspective of the patient and the broader societal impact. It should be noted that the cost of visiting a psychiatrist during normal operating hours was lower than the private hospital because the KCMH is a public hospital that receives some taxpayer support. Furthermore, direct medical costs were reported in cumulative terms, which was annualized based on the number of months observed in this study.

#### Medical comorbidities

In the present study, the comorbidities of MDD patients were assessed using the Charlson comorbidity index (CCI). This index, which was derived from data extracted from medical charts by a psychiatrist, consists of 21 specific comorbidities: myocardial infarction, congestive heart failure, peripheral vascular disease or bypass, cerebrovascular disease or transient ischemic disease, hemiplegia, pulmonary disease/asthma, diabetes, diabetes with end organ damage, renal disease, mild liver disease, severe liver disease, gastric or peptic ulcer, cancer (lymphoma, leukemia, solid tumor), metastatic solid tumor, dementia or Alzheimer's disease, rheumatic or connective tissue disease, HIV or AIDS, hypertension, skin ulcers/cellulitis, depression, and warfarin usage. Each comorbidity is assigned a score of 1, 2, 3 or 6 based on the associated risk of mortality. MDD patients 50 years old or older receive additional points; one to three points, in the scoring system. In fact, the analysis of the present study focused primarily on the direct medical costs associated with MDD and did not include direct medical costs specifically related to the comorbidities assessed by the CCI. The decision to exclude costs was made to maintain the scope and focus. However, it is important to mention the presence of comorbidities among MDD patients in a hospital setting as a supplementary aspect of the study. Comorbidities are common in individuals with MDD and can significantly impact their overall health status, treatment outcomes, and healthcare utilization.

#### Direct nonmedical costs

The tangible expenses associated with MDD or its treatment, but not medical activities, are referred to as direct nonmedical costs. In this study, direct nonmedical costs included payment for caregiving services and transportation to the hospital (including a parking fee if driving their own vehicle). Both costs were obtained from the cross-sectional questionnaire. The route from the MDD patient’s home to the hospital was estimated using Google Maps. Moreover, when estimating the transportation cost for individuals driving their own vehicles, it was assumed that the fuel cost would be 35 Baht per liter, and the fuel consumption rate would be 10 km per liter.

#### Indirect costs

The opportunity cost of MDD and its treatment is related to the intangible expenses of MDD. In this research, we evaluated the indirect costs of the productivity losses of patients and their unpaid caregivers due to MDD, and unpaid help to assist patients in their daily activities. For patients and their unpaid caregivers who were currently employed in the formal or informal sectors of the labor market, the Work Productivity and Activity Impairment Questionnaire: Depression version 2.0, patient version (WPAI:D) and Work Productivity and Activity Impairment Questionnaire: Mood & Mental State, caregiver version (WPAI:MM-CG), were used to measure absenteeism and presenteeism of MDD patients and their unpaid caregivers due to the patient's MDD symptoms. Absenteeism was defined as the percentage of work or study time missed due to MDD and TRD. Presenteeism was indicated as a percentage of impairment while working or studying due to MDD and TRD. The Class Impairment Questionnaire (CIQ) was used to assess productivity loss in MDD patients or unpaid caregivers who were university students. Productivity loss was calculated in terms of lost working or studying hours and converted to financial annual terms.

To collect the time spent on unpaid caregiving activities instead of leisure time or part-time work, we used the Family Experiences Interview Schedule (FEIS), which compiles eight daily activities that an unpaid caregiver does for the patient. The questionnaire was a five-point Likert scale that we transformed to 0, 0.5, 1.5, 4.5, and 7 h per week. The Thai minimum wage (i.e., Bangkok’s rate in 2022) was used as a multiplier of the opportunity cost of time spent. Notably, we left out the indirect cost of job losses caused by MDD and TRD because, at the time of data collection, the Thai economy was experiencing a slowdown due to the COVID-19 pandemic. Consequently, there was an increased rate of layoffs and unemployment in Thailand; therefore, it was challenging to determine the true cause of the patient’s unemployment.

#### Health insurance systems in Thailand

In Thailand, the healthcare insurance landscape comprises two primary categories: public health insurance and private health insurance. The public health insurance system is integrated into the national healthcare framework and encompasses three major subsystems: the civil servant healthcare scheme, the social security scheme, and the universal healthcare scheme. Each subsystem provides distinct levels of coverage tailored to different segments of the Thai population. The universal healthcare scheme functions as the principal health insurance system, extending coverage to the general populace beyond the purview of the civil servant and social security schemes. Under this scheme, individuals are entitled to medical treatment at state hospitals where they are registered without incurring any expenses. The civil servant healthcare scheme exclusively caters to civil servants and their families, while the social security scheme caters to employees in the formal business sector. Importantly, individuals can only be enrolled in a single public health insurance subsystem. Within the public health insurance framework, individuals are exempt from paying for medical services when seeking care at the state hospitals where they are registered under the public insurance. However, if they seek treatment at private hospitals or other public hospitals where they lack the eligibility for public insurance, they are liable for the associated costs. As an additional measure, individuals have the option to individually acquire private health insurance to augment their coverage specifically for private hospital services.

In the present study, patients diagnosed with MDD were surveyed regarding whether they personally paid for their medical expenses or used public or private health insurance coverage. When health insurance was used, medical expenses were typically covered, with the exception of medications that were not registered on the national drug list.

#### Other independent variables

The selection of characteristic factors was based on the literature review of previous studies. We collected demographic, socioeconomic, and behavioral information on MDD patients and their caregivers from the cross-sectional survey. These data included their age, height, weight, marital status, degree of educational attainment, income, COVID-19 infection, and the effects of lockdown measures on MDD treatments in Thailand, as well as their use of alcohol, tobacco, and other drugs.

### Statistical analyses

Data analysis was performed with SPSS version 29.0 software. Descriptive data were presented as frequency, percentages, means, median, standard deviation (SD), and interquartile range (IQR). We calculated the economic burden of TRD and non-TRD based on microdata analysis. Mean differences and associations between characteristic variables and response/resistance to MDD treatment were analyzed using Pearson Chi-square test and Fisher’s exact test for dichotomous variables, and Student’s T-test for continuous variables. A p-value less than 0.05 was considered statistically significant.

## Results

The findings of the study indicated that out of the total 500 antidepressant-treated MDD patients included in the analysis, 98 patients (19.6%) had a history of using two or more antidepressants. These patients did not respond to treatment on at least two occasions during the observation period, meeting the criteria for TRD. Table 1 listed the typical characteristics of MDD patients and their unpaid caregivers. The majority of the MDD patients (77.4%) was female. The respondents' mean age was 35.1 years (SD = 13.8), and the mean age of MDD onset was 30.6 years (SD = 12.7). The body mass index (BMI) of 55.4% of the MDD patients was in the underweight-to-normal range (< 25.0). About one-third of the MDD patients (36.0%) were of working age and employed full-time. The study's findings indicate that 57.2% of MDD patients were responsible for self-funding their MDD treatment at the hospital. This implied a significant portion of MDD patients did not have private health insurance coverage, which led them to bear the financial burden of their MDD treatment. Additionally, the eligibility for public health insurance was limited to other state hospitals, indicating that these patients were unable to benefit from public health insurance coverage at the specific hospital where the study took place. In terms of unpaid caregivers of MDD patients, 58.2% were female, and 32.2% were spouses or partners of MDD patients.

**Table 1:** Baseline Characteristics of MDD Patients and Their Unpaid Caregivers (*N* = 500)

Variables	All (*N* = 500)	TRD (*n* = 98)	Non-TRD (*n* = 402)	*p*-value	Effect sizes	Variables	All (*N* = 500)	TRD (*n* = 98)	Non-TRD (*n* = 402)	*p*-value	Effect sizes
**MDD patients**						**BMI**					
**Biological sex**						Mean (SD)	24.9 (5.7)	23.1 (4.5)	25.4 (5.9)	< 0.001*	-0.402
Female	387 (77.4)	78 (79.6)	309 (76.9)	0.563	1.174	< 25.0	277 (55.4)	67 (68.4)	210 (52.2)	0.003*	
Male	113 (22.6)	20 (20.4)	93 (23.1)			25.0–29.9 (Overweight)	133 (26.6)	24 (24.5)	109 (27.1)		
**Gender**						≥ 30.0 (Obesity)	90 (18.0)	7 (7.1)	83 (20.6)		
Female	357 (71.4)	70 (71.4)	287 (71.4)	0.865		**Marital status**					
Male	98 (19.6)	18 (18.4)	80 (19.9)			Single	335 (67.0)	73 (74.5)	262 (65.2)	0.220	
LGBTQIA +	45 (9.0)	10 (10.2)	35 (8.7)			Married/De-Facto relationship	126 (25.2)	20 (20.4)	106 (26.4)		
**Age (years)**						Separated	11 (2.2)	3 (3.1)	8 (2.0)		
Mean (SD)	35.1 (13.8)	32.2 (12.7)	36.0 (14.1)	0.010*	-0.276	Divorced	19 (3.8)	2 (2.0)	17 (4.2)		
Median (IOR)	30.0 (22.0)					Widowed	9 (1.8)	0 (0.0)	9 (2.2)		
Range	18–75					**Employment status**					
18–25	168 (33.6)	41 (41.8)	127 (31.6)	0.153		Full-time	180 (36.0)	36 (36.7)	144 (35.8)	0.502	
26–35	145 (29.0)	29 (29.6)	116 (28.9)			Part-time/freelance	26 (5.2)	1 (1.0)	25 (6.2)		
36–50	93 (18.6)	15 (15.3)	78 (19.4)			Business owner	36 (7.2)	5 (5.1)	31 (7.7)		
51–65	83 (16.6)	16 (13.3)	70 (17.4)			Homemaker	57 (11.4)	10 (10.2)	47 (11.7)		
> 65	11 (2.2)	0 (2.2)	11 (2.7)			Unemployed, seeking work	58 (11.6)	14 (14.3)	44 (10.9)		
**Age of MDD onset (years)**						Retired	24 (4.8)	5 (5.1)	19 (4.7)		
Mean (SD)	30.5 (12.7)	28.1 (11.6)	31.1 (12.9)	0.035*	-0.238	University students	97 (19.4)	24 (24.5)	73 (18.2)		
Median (IOR)	26.0 (17.0)					Other	22 (4.4)	3 (3.1)	19 (4.7)		
						**Income (Baht per month)**					
**Number of Antidepressant’s lines**						Mean (SD)	17,766.7 (1,523.3)	16,526.8 (26,646.9)	18,068.2 (35,658.2)	0.689	-0.045
1 line	363 (72.6)	0 (0.0)	363 (90.3)	< 0.001*		**History of self-harm**					
2 lines	108 (21.6)	69 (70.4)	39 (9.7)			Yes	97 (19.4)	35 (35.7)	62 (15.4)	< 0.001*	3.047
3 lines	25 (5.0)	25 (25.5)	0 (0.0)			No	403 (80.6)	63 (64.3)	340 (84.6)		
≥ 4 lines	4 (0.8)	4 (4.1)	0 (0.0)			**History of suicide attempts**					
**Daily smoking behavior**						Yes	58 (11.6)	25 (25.5)	33 (8.2)	< 0.001*	3.829
Yes	22 (4.4)	10 (10.2)	12 (3.0)	0.004^a^	3.693	No	442 (88.4)	73 (74.5)	369 (91.8)		
No	478 (95.6)	88 (89.8)	390 (97.0)								
**Daily alcohol behavior**											
Yes	10 (2.0)	1 (1.0)	9 (2.2)	0.695^a^	0.450						
No	490 (98.0)	97 (99.0)	393 (97.8)								
**Substance use**						**Highest educational attainment**					
Yes	12 (2.4)	4 (4.1)	8 (2.0)	0.263^a^	2.096	No schooling or primary education	20 (4.0)	1 (1.0)	19 (4.7)	0.510	
No	488 (97.6)	94 (95.9)	394 (98.0)			Lower secondary education	16 (3.2)	2 (2.0)	14 (3.5)		
**Hired caregiver for MDD patient**						Upper secondary education	36 (7.2)	7 (7.1)	29 (7.2)		
Yes	7 (1.4)	3 (3.1)	4 (1.0)	0.140^a^	0.318	Post-secondary education	21 (4.2)	4 (4.1)	17 (4.2)		
No	493 (98.6)	95 (96.9)	398 (99.0)			Bachelor’s or equivalent level	218 (43.6)	41 (41.8)	177 (44.0)		
**Comorbidities from CCI**						Master’s and Doctoral levels	69 (13.8)	18 (18.4)	51 (12.7)		
Mean score of CCI (SD)	1.6 (1.3)	1.3 (0.7)	1.6 (1.4)	0.001*	-0.265	Currently studying	120 (24.0)	25 (25.5)	95 (25.5)		
Myocardial infarction	1 (0.2)	0 (0.0)	1 (0.2)	1.000^a^		**Healthcare coverage for depression**					
Congestive heart failure	1 (0.2)	0 (0.0)	1 (0.2)	1.000^a^		Government healthcare coverage					
Peripheral vascular disease or bypass	0 (0.0)	0 (0.0)	0 (0.0)			-Under universal coverage scheme	118 (23.6)	31 (31.6)	87 (21.6)	0.189	
Cerebrovascular disease	8 (1.6)	0 (0.0)	8 (2.0)	0.365^a^		-Under social security scheme	41 (8.2)	9 (9.2)	32 (8.0)		
Hemiplegia, pulmonary disease/asthma	1 (0.2)	0 (0.0)	1 (0.2)	1.000^a^		-Under Thai Civil Servant Scheme	50 (10.0)	10 (10.2)	40 (10.0)		
Diabetes	9 (1.8)	1 (1.0)	8 (2.0)	1.000^a^		Private insurance	5 (1.0)	0 (0.0)	5 (1.2)		
Diabetes with end organ damage	1 (0.2)	0 (0.0)	1 (0.2)	1.000^a^		Self-funded	286 (57.2)	48 (49.0)	238 (59.2)		
Renal disease	3 (0.6)	0 (0.0)	3 (0.7)	1.000^a^		**Transportation to hospital**					
Mild liver disease	5 (1.0)	0 (0.0)	5 (1.2)	0.588^a^		Public transportation	230 (46.0)	35 (35.7)	195 (48.5)	0.023*	0.590
Severe liver disease	2 (0.4)	0 (0.0)	2 (0.5)	1.000^a^		Take taxi	91 (18.2)	21 (21.4)	70 (17.4)	0.853	1.294
Gastric or peptic ulcer	5 (1.0)	1 (1.0)	4 (1.0)	1.000^a^		Drive own vehicle	218 (43.6)	47 (48.0)	171 (42.5)	0.942	1.245
Cancer	15 (3.0)	3 (3.1)	12 (3.0)	1.000^a^		Walk	11 (2.2)	1 (1.0)	10 (2.5)	0.700^a^	0.404
Metastatic solid tumor	2 (0.4)	0 (0.0)	2 (0.5)	1.000^a^		**Caregiver’s gender**					
Dementia or Alzheimer's disease	0 (0.0)	0 (0.0)	0 (0.0)			Female	291 (58.2)	237 (59.0)	54 (55.1)	0.758	
Rheumatic or connective tissue disease	5 (1.0)	0 (0.0)	5 (1.2)	0.588^a^		Male	192 (38.4)	152 (37.8)	40 (40.8)		
HIV or AIDS	4 (0.8)	0 (0.0)	4 (1.0)	1.000^a^		LGBTQIA +	17 (3.4)	13 (3.2)	4 (4.1)		
Hypertension	15 (3.0)	0 (0.0)	15 (3.7)	0.051^a^		**Caregiver’s age (years)**					
Skin ulcers/cellulitis	1 (0.2)	1 (1.0)	0 (0.0)	0.196^a^		Mean (S.D.)	42.2 (0.7)	42.0 (14.8)	42.2 (15.6)	0.913	-0.012
Warfarin usage	2 (0.4)	0 (0.0)	2 (0.5)	1.000^a^		Median (IOR)	40.0 (28)				
**Unpaid caregiver**						**Caregiver’s employment status**					
**Relationship with MDD patient**						Full-time	227 (45.4)	50 (51.0)	177 (44.0)	0.889	
Spouse	161 (32.2)	31 (31.6)	130 (32.3)	0.481		Part-time/freelance	19 (3.8)	5 (5.1)	14 (3.5)		
Child (daughter/son)	40 (8.0)	3 (3.1)	37 (9.2)			Business owner	46 (9.2)	9 (9.2)	44 (10.9)		
Siblings	73 (14.6)	15 (15.3)	58 (14.4)			Homemaker	54 (10.8)	9 (9.2)	45 (11.2)		
Uncle/Aunt	5 (1.0)	1 (1.0)	4 (1.0)			Unemployed, seeking work	21 (4.2)	2 (2.0)	19 (4.7)		
Grandchild	6 (1.2)	1 (1.0)	5 (1.2)			Retired	48 (9.6)	9 (9.2)	39 (9.7)		
Friend/ Neighbor/ Acquaintance	84 (16.8)	18 (18.4)	66 (16.4)			University students	53 (10.6)	9 (9.2)	44 (10.9)		
Parents	124 (24.8)	27 (27.6)	97 (24.1)			Other	32 (6.4)	5 (5.1)	27 (6.6)		
Other	7 (1.4)	2 (2.0)	5 (1.2)								

The results of Pearson Chi-square, Fisher’s Exact Test, and Independent T-Test were reported in the table. Crude Odds Ratio was reported as the effect sizes of the statistical results of Pearson Chi-square and Fisher’s Exact Test. Cohen’s *d* was reported as the effect sizes of the statistical results of Independent T-test. Crude Odds Ratio was available for the 2 × 2 table. ^a^ referred to the statistical result of Fisher’s Exact Test and * indicated the statistical significance at 0.05

Regarding the continuous variables of MDD patients, Table 1 presents the differences in baseline characteristics and other personal factors between MDD patients with TRD and those with non-TRD. The analysis revealed statistically significant differences in several variables. MDD patients with TRD had a statistically significant lower mean age (*p* = 0.010, Cohen’s *d* = -0.276), lower mean age of onset of MDD (*p* = 0.035, Cohen’s *d* = -0.238), and lower mean BMI (*p* < 0.001, Cohen’s *d* = -0.402) compared to MDD patients with non-TRD, with a small effect size. Additionally, we found an insignificant difference in average income per month between two groups of MDD patients. For dichotomous variables of MDD patients, Table 1 shows the bivariate analysis of characteristic variables and TRD criteria. The result indicated that the patient’s history of self-harm (*p* < 0.001, Crude Odds Ratio (OR) = 3.047), the occurrence of suicide attempts (*p* < 0.001, Crude OR = 3.829), and frequent smoking behavior (*p* = 0.004, Crude OR = 3.693) were significantly associated with TRD.

The mean Charlson Comorbidity Index (CCI) was estimated to be 1.3 (SD = 0.7) for MDD patients with TRD and 1.6 (SD = 1.4) for MDD patients with non-TRD in terms of medical comorbidities. Among MDD patients with TRD, comorbidities such as cancer were present in 3.1% of patients, diabetes in 1.0%, gastric or peptic ulcer in 1.0%, and skin ulcers/cellulitis in 1.0%. On the other hand, MDD patients with non-TRD had comorbidities including hypertension in 3.7% of patients, cancer in 3.0%, cerebrovascular disease in 2.0%, diabetes in 2.0%, mild liver disease in 1.2%, rheumatic or connective tissue disease in 1.2%, gastric and peptic ulcer in 1.0%, HIV or AIDS in 1.0%, renal disease in 0.7%, severe liver disease in 0.5%, metastatic solid tumor in 0.5%, warfarin usage in 0.5%, myocardial infarction in 0.2%, congestive heart failure in 0.2%, hemiplegia, pulmonary disease/asthma in 0.2%, diabetes with end organ damage in 0.2% (Table 1).

**Fig. 2:**
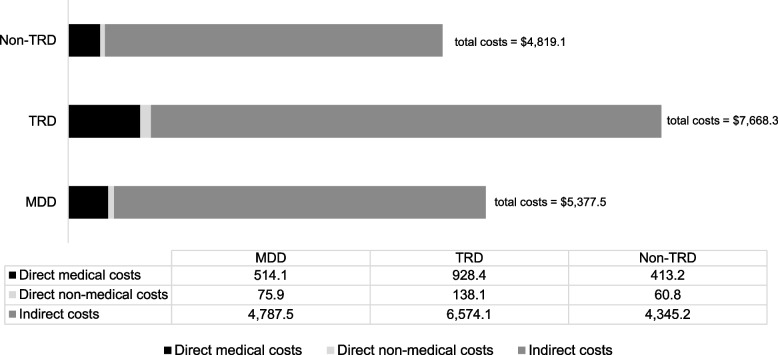
The Comparison of Economic Costs TRD and non-TRD among MDD Patients in the Approach of Societal Perspective (Without Health Insurance Applications)

According to the result illustrated in Table 2, the study found that the average total annual costs, average annual direct medical costs, and average annual indirect costs of TRD were significantly higher compared to those of non-TRD among patients diagnosed with MDD, but the average annual direct non-medical costs were not statistically different between the two groups of MDD patients. The direct medical costs in this study were categorized into two approaches, one with health insurance coverage application which capturing the costs that MDD patients paid for their medical treatment (MDD patients’ perspective), and the other without health insurance coverage application which focusing on the actual costs incurred by MDD patients in the healthcare system (the societal view including the healthcare provider’s costs). The average annual economic cost of MDD covered by health insurance was 183,478.33 Baht ($5,096.62) per individual. There were three average total direct costs: 8,396.03 Baht ($233.22) for medical expenses, 2,731.93 Baht ($75.89) for nonmedical expenses, and 172,350.38 Baht ($4,787.51) for indirect expenses. In comparison to the societal perspective of direct medical costs or without health insurance application, the study found that the average annual direct medical cost was 18,509.03 Baht ($514.14), while the average annual cost of MDD per person was 193,591.33 Baht ($5,377.54) (Fig. 2). These findings indicate that the actual direct medical costs incurred by MDD patients were significantly higher than the costs that patients paid out of pocket. Specifically, the average annual direct medical cost incurred by MDD patients was more than double the cost paid by patients themselves (18,509.03 Baht vs. 8,396.03 Baht) (Table 2).

**Table 2:** Elaboration of Total Costs, Direct Medical Costs, Direct Non-Medical Costs, and Indirect of MDD, TRD, and Non-TRD (*N* = 500)

**Variables**	**Without insurance application**					**With insurance application**				
	**All MDD patients** **(*N* = 500)**	**TRD** **(*n* = 98)**	**Non-TRD** **(*n* = 402)**	***p***	***d***	**All MDD patients** **(*N* = 500)**	**TRD** **(*n* = 98)**	**Non-TRD** **(*n* = 402)**	***p***	***d***
**1. Total costs (Baht) (1.1 + 1.2 + 1.3)**	**193,591.33** **(10,623.67)**	**276,059.97** **(277,548.88)**	**173,487.04** **(222,530.15)**	< 0.001*	**0.438**	**183,478.33** **(10,579.53)**	**256,867.58** **(283,391.76)**	**165,587.42** **(220,399.87)**	0.003*	**0.390**
	**$5,377.54**	**$7,668.33**	**$4,819.08**			**$5,096.62**	**$7,135.21**	**$4,599.65**		
Median (IQR) (Baht)	111,537.66 (185,638.99)	189,737.09 (316,822.60)	107,163.65 (169,129.95)			106,221.31 (182,485.04)	143,174.04 (338,343.84)	101,632.99 (169,367.66)		
Min (Baht)	1,823.81	13,098.98	1,823.81			76.19	3454.80	76.19		
Max (Baht)	2,203,833.75	1,475,939.14	2,203,833.75			2,168,752.69	1,475,939.14	2,168,752.69		
**1.1 Total direct medical cost (Baht)**	**18,509.03** **(1,057.67)**	**33,421.56** **(30,562.65)**	**14,873.63** **(20,062.03)**	< 0.001*	**0.825**	**8,396.03** **(734.25)**	**14,229.17** **(25,008.58)**	**6,974.02** **(13,183.57)**	0.006*	**0.448**
	**$514.14**	**$928.38**	**$413.16**			**$233.22**	**$395.25**	**$193.72**		
Median (IQR) (Baht)	9,085.72 (22,418.33)	22,261.05 (32,288.72)	6,227.33 (19,526.73)			2,155.05 (8,636.89)	0.00 (17,333.95)	2,169.48 (6,638.30)		
Min (Baht)	792.86	2,826.05	792.86			0.00	0.00	0.00		
Max (Baht)	183,409.01	152,667.54	183,409.01			118,262.83	118,262.83	107,571.54		
a. Outpatient visits (Baht)	787.44 (22.41)	1,222.70 (798.42)	681.33 (317.27)	< 0.001*	1.195	448.19 (23.10)	612.89 (819.69)	408.04 (412.24)	0.018*	0.396
	$21.87	$33.96	$18.93			$12.45	$17.02	$11.33		
b. Inpatient visits (Baht)	895.09 (248.85)	3,379.99 (11,127.82)	289.32 (2,588.69)	0.007*	0.569	360.01 (134.86)	1090.58 (5,075.49)	181.91 (2,218.59)	0.086	0.303
	$24.86	$93.89	$8.04			$10.00	$30.29	$5.05		
c. Emergency visits (Baht)	29.66 (8.65)	107.40 (401.90)	10.70 (75.20)	0.020*	0.510	362.03 (134.65)	179.84 (942.94)	406.44 (3324.96)	0.505	-0.075
	$0.82	$2.98	$0.30			$10.06	$5.00	$11.29		
d. Medications (Baht)	16,482.86 (932.67)	27,886.74 (23,386.71)	13,702.80 (19,219.66)	< 0.001*	0.706	7,528.53 (673.91)	12,297.20 (21,451.38)	6,366.01 (12,819.85)	0.010*	0.398
	$457.86	$774.63	$380.63			$209.13	$341.59	$176.83		
e. Laboratory tests (Baht)	313.99 (36.90)	824.72 (1,405.87)	189.48 (538.37)	< 0.001*	0.808	155.36 (26.97)	378.25 (1,042.00)	101.03 (418.22)	0.011*	0.467
	$8.72	$22.91	$5.26			$4.32	$10.51	$2.81		
f. Psychotherapy and occupational therapy (Baht)	0.0 (0.0)	0.0 (0.0)	0.0 (0.0)	-	-	0.0 (0.0)	0.0 (0.0)	0.0 (0.0)	-	-
	$0	$0	$0			$0	$0	$0		
**1.2 Total direct non-medical cost (Baht)**	**2,731.93** **(597.77)**	**4,971.17** **(21,386.83)**	**2,186.91** **(10,495.91)**	**0.213**	**0.209**					
	**$75.89**	**$138.09**	**$60.75**							
Median (IQR) (Baht)	645.77 (932.95)	1040.67 (2,299.65)	588.58 (764.71)							
Min (Baht)	0.00	0.00	0.00							
Max (Baht)	181,297	181,297.27	126,276.43							
a. Transportation to hospital (Baht)	1,565.53 (270.40)	2,938.52 (11,487.64)	1,230.82 (3,605.08)	0.149	0.284					
	$43.49	$81.63	$34.19							
b. Paid caregiver services (Baht)	1,164.40 (535.07)	2,032.65 (18,213.91)	955.22 (9,882.25)	0.425	0.090					
	$32.34	$56.46	$26.53							
**1.3 Total indirect cost (Baht)**	**172,350.38** **(10,339.25)**	**236,667.24** **(269,675.91)**	**156,427.36** **(218,229.10)**	0.006*	**0.355**					
	**$4,787.51**	**$6,574.09**	**$4,345.20**							
Median (IQR) (Baht)	95,902.56 (184,178.09)	132,969.84 (299,724.64)	89,476.40 (173,237.90)							
Min (Baht)	0.00	2,111.20	0.00							
Max (Baht)	2,168,444.80	1,454,778.40	2,168,444.80							
a. Productivity loss of patient (Baht)	78,954.14 (7,099.56)	112,609.81 (221,176.19)	70,749.52 (138,504.13)	0.076	0.265					
	$2,193.17	$3,128.05	$1,965.26							
b. Productivity loss of an unpaid caregiver due to the MDD symptom of patient (Baht)	61,044.21 (7481.22)	89,845.63 (168,131.04)	54,022.97 (166,533.40)	0.060	0.215					
	$1,695.67	$2,495.71	$1,500.64							
c. Unpaid caregiving time cost that was spent helping patients in daily living (Baht)	32,352.03 (1,146.89)	35,211.80 (26,138.83)	31,654.87 (25,507.93)	0.219	0.139					
	$898.67	$978.11	$879.30							

The result of the Student’s T-test was presented in the table. Cohen’s *d* (mentioned as “*d*” in the table) presented the effect size of the result. Standard deviation is shown in parentheses and * indicated the statistical significance at 0.05

Additionally, when considering the perspective of society which included the actual medical costs from the healthcare provider (without health insurance application), the study found a significant difference in actual direct medical expenses between the two groups, with a large effect size (mean difference = 18,547.93 Baht ($515.22), *p* < 0.001, Cohen's *d* = 0.825). While considering the perspective of MDD patients (with insurance application), the study also found a significant difference in direct medical costs between the two groups of MDD patients, with a small effect size (mean difference = 7,255.15 Baht ($201.53), *p* = 0.006, Cohen’s *d* = 0.448). The findings imply that MDD patients with TRD had significantly greater direct medical expenses than MDD patients without TRD. When considering the actual direct medical expenses incurred by MDD patients (without health insurance applications), the difference became more evident. According to social viewpoint, there was a bigger gap between TRD and non-TRD, indicating a significant financial burden associated with TRD in terms of healthcare costs. Furthermore, it was observed during the study period that MDD patients did not receive occupational therapy or psychotherapy as part of their treatment. The absence of these therapeutic interventions raises important considerations and implications, which will be discussed in the subsequent section of the study.

Based on an anticipated sample size of 500 dyads of MDD patients and their unpaid caregivers, Table 3 shows the total economic costs of MDD, TRD, and non-TRD from a social perspective including actual medical costs from the healthcare provider. In total, it was calculated that MDD, TRD, and non-TRD each cost 96.80 million baht ($2.69 million), 27.05 million baht ($0.75 million), and 69.74 million baht ($1.94 million).

**Table 3:** The Cumulative of the Economic Costs of Major Depressive Disorder, Treatment-Resistant Depression, and Treatment-Responsive Depression (*N* = 500) by Given 1 USD = 36 Baht

Costs (Million baht per year)	Without insurance application			With insurance application		
	**MDD patients (*N* = 500)**	**TRD** **(*n* = 98)**	**Non-TRD** **(*n* = 402)**	**MDD patients** **(*N* = 500)**	**TRD** **(*n* = 98)**	**Non-TRD** **(*n* = 402)**
The economic costs (million Baht)	96.80	27.05	69.74	91.74	25.17	66.57
	$2.69 M	$0.75 M	$1.94 M	$2.55 M	$0.70 M	$1.85 M
Direct medical costs (million Baht)	9.25	3.28	5.98	4.20	1.39	2.80
	$0.26 M	$0.09 M	$0.17 M	$0.12 M	$0.04 M	$0.08 M
Direct non-medical costs (million Baht)	1.37	0.49	0.88	1.37	0.49	0.88
	$0.04 M	$0.01 M	$0.02 M	$0.04 M	$0.01 M	$0.02 M
Indirect costs (million Baht)	86.18	23.19	62.88	86.18	23.19	62.88
	$2.39 M	$0.64 M	$1.75 M	$2.39 M	$0.64 M	$1.75 M

## Discussion

The present study aimed to investigate the prevalence and economic burden of TRD within the population of individuals diagnosed with MDD in a hospital setting situated in Thailand. The study findings revealed that the prevalence of TRD among MDD patients in a Thai tertiary public hospital was 19.6%. In terms of the economic burden, from the perspective of society, the annualized economic cost of TRD was determined to be 276,059.97 baht per person ($7,668.33), which was significantly higher than the cost of MDD patients without current TRD conditions (173,487.04 baht or $4,819.08). The aggregated economic costs of MDD, considering 500 MDD patients and unpaid caregivers, were estimated to be 96.8 million baht annually ($2.69 million). Within this total, the economic cost of TRD accounted for 27.05 million baht (98 dyads of MDD patients and unpaid caregivers), while the economic cost of non-TRD accounted for 69.74 million baht (402 dyads of MDD patients and unpaid caregivers). Additionally, the study identified several factors that were significantly associated with TRD among individuals with MDD. These factors included a lower mean age, a lower mean age of onset of MDD, a lower body mass index (BMI), the presence of suicide attempts and self-harm, and frequent smoking behavior. However, the study did not find a significant association between TRD and biological sex of MDD patients, frequent alcohol behavior, or substance use among MDD patients.

The findings of the present study indicated a statistically significant difference in the economic burden between TRD and non-TRD among MDD patients in a Thai tertiary hospital. Specifically, the study revealed that TRD was associated with higher direct medical costs and indirect costs compared to non-TRD. The higher direct medical costs observed in patients with TRD suggested increased healthcare resources. These findings highlight the increased healthcare needs and utilization among MDD patients with TRD compared to those non-TRD. In addition, the study revealed higher indirect costs associated with TRD, indicating a significant loss in work or study among MDD patients with TRD and their unpaid caregivers. This suggests that TRD not only imposes a financial burden on the healthcare system, but also has a substantial impact on the productivity and well-being of individuals with TRD and their unpaid caregivers. Moreover, the study found no significant differences in the proportion of men and women between individuals with TRD and those without TRD, suggesting that both male and female individuals with MDD have a similar likelihood of developing TRD. However, it was also found that MDD patients with TRD had a lower mean age of onset of MDD compared to those with non-TRD. That is, individuals who experienced the onset of MDD at a younger age may have a poorer prognosis in terms of resistance to treatment. Furthermore, the study found that the occurrences of suicide attempts and self-harm were significantly related to TRD. This suggests that MDD patients with TRD experienced the increased risk of engaging in suicide attempts and self-harming behaviors.

The present study revealed that 19.6% of antidepressant-treated MDD patients met the criteria for TRD. This finding is consistent with prior international investigations, which have indicated a considerable variation in TRD prevalence, ranging from 12 to 55% [30]. The discrepancies in reported prevalence can be attributed to differences in the methodology and targeted population, the definition of TRD utilized, and the specific diagnostic criteria employed to identify patients with depression [31]. The present study's findings on TRD among antidepressant-treated MDD patients closely align with two prior investigations. Firstly, Fife et al. (2017) conducted a study utilizing data from Taiwan's National Health Insurance Research database in 2005, which reported a TRD prevalence of 21%. Another study was carried out at Mexican public and private psychiatric reference sites which reported a TRD prevalence of 21% [20] In both cases, the criteria for TRD included MDD treatment with two or more antidepressants and a lack of adequate responses to these treatments. Indeed, it is crucial to acknowledge that variations in the prevalence calculations between the Taiwanese and Latin American studies were not solely attributable to differences in the countries and data collection settings of research but also stem from discrepancies in the denominators used. Specifically, the Taiwanese study considered patients with pharmaceutically treated depression (PTD) as the denominator, while the Latin American study included patients diagnosed with MDD as the denominator. However, the finding was contrary to some previous studies.

The current investigation yielded a lower prevalence of TRD than the rates reported in various other regions, including Israel's Maccabi healthcare services database (24.4% in 2016–2018) [32], Poland's outpatient private and public clinics (25.2% in 2020–2021) [33], the United States (30.9%) [26], and UK primary care settings (55%) [31]. Each of these earlier studies employed distinct criteria for identifying TRD, such as the number of lines of treatment indicated in prescriptions, reduction in BDI score, or adherence to antidepressant medication. Comparatively, the present study demonstrated a higher prevalence of TRD than some previous investigations, such as those conducted in South Korea (15.3%) [34] and the United States (5.8–6.0%) [35]. Another study in South Korea reported a TRD prevalence of 4.2% among patients with pharmaceutically treated depression (PTD) in 2012, identified through reviewing codes and prescriptions of diagnostic medication using the Health Insurance Review and Assessment [36]. The variations in TRD prevalence observed across different studies highlight the complex and multifaceted nature of this condition. However, drawing definitive conclusions solely based on these prevalence rates requires careful consideration of various contributing factors.

Generalizing the prevalence approach used in this study to the Thai population may likely result in a higher prevalence of TRD among antidepressant-treated MDD patients compared to the overall Thai population. Several factors contribute to this potential difference, including the setting of the public hospital, which often handles more severe cases of MDD, and the denominator used in the prevalence calculation, which focusses on antidepressant-treated MDD patients, narrowing down the target population.

Results show that the mean age of TRD patients, age of onset of MDD, and BMI were considerably lower than those of the non-TRD patients. The outcome was incongruent from findings of Gronemann, et al. (2020) that found MDD patients of older age had a significant risk of TRD. We found no consistency in the association between age of onset of depression and TRD. Regarding the relationship between BMI and TRD, results contradict Warrings, et al. (2021) that found higher BMI to be a risk factor for TRD due to higher adequate doses of antidepressants required [37]. In addition, a significant difference in the MDD between men and women was also found in our study. Particularly, more women than men had MDD, TRD, and non-TRD, according to the study. This concurs with general trends observed in prior studies, which indicates that women are more likely than men to experience MDD [38–44].

However, our study revealed a significant association between the history of self-harm and suicide attempts and TRD. These findings align with existing empirical evidence that indicates that patients with TRD exhibit higher rates of suicide attempts and self-harm compared to non-TRD patients [45–47]. Previous research explained that TRD was associated with more severe depression, which increased the probability of experiencing suicidal thoughts and engaging in suicide attempts [48, 49]. Furthermore, the link between TRD and suicide attempts was explored by identifying different neuronal endophenotypes. Previous studies provided the results of the underlying neural mechanisms that may contribute to this association [50, 51]. However, it is important to acknowledge that in our present study, we did not conduct a survey to assess the present symptoms and severity of depression among the participants. Additionally, we did not collect laboratory results related to neurobiological markers or neuronal endophenotypes. The absence of these assessments and data points limits our ability to make direct inferences about the current symptomatology and neurobiological profiles of study participants. Consequently, we cannot draw definitive conclusions regarding the immediate relationship between TRD, present depressive symptom severity, and specific neurobiological markers. Furthermore, our study revealed a significant relationship between habitual smoking behavior and patients with MDD who have TRD. This finding is consistent with previous research [52, 53]. In the study by Korchia et al. (2022) conducted on French cohorts of severe psychiatric disorders, tobacco smoking was associated with unfavorable outcomes in TRD, particularly in women. The researchers found that smoking was associated with increased suicidality and a higher prescription rate for third- or fourth-line TRD treatments in MDD patients who smoked.

In 2022, when considering the direct medical cost of MDD from the perspective of patients, the annualized economic costs of MDD, TRD, and non-TRD were 183,478.33 baht ($5,096.62), 256,867.58 baht ($7,135.21), and 165,587.42 baht per person ($4,599.65), respectively. Annual economic costs of MDD were 91.74 million baht ($91.74 million) under health insurance implementation (patient’s perspective) and 96.8 million baht ($96.80 million) under no insurance implementation (societal perspective) if aggregated into a cumulative term of 500 dyads of MDD patients and their unpaid caregivers. Public and private health insurance systems have contributed to providing free or discounted medical care to individuals diagnosed with MDD. This equitable access to mental health services is particularly beneficial for the lower socioeconomic class [54]. However, it is important to acknowledge that the insurance claims may introduce distortions in the economic cost estimation of MDD, TRD, and non-TRD from a societal perspective. Specifically, the direct medical costs incurred by these patients could be potentially lower than they would be in the absence of insurance coverage. As a result, caution is warranted when interpreting the economic burden of MDD in the broader societal context, taking into account the impact of insurance claims on the cost estimation.

The main source of the economic costs of MDD, TRD, and non-TRD was from indirect costs, especially productivity loss, absenteeism and presenteeism. This is in line with previous international research showing that indirect costs from lost productivity and jobs were substantially higher than direct medical costs [27, 28]. In 2017, Sousa, et al. (2022) estimated the economic cost of TRD in Portugal using national data and found that the direct cost accounted for € 3.1 billion whereas the indirect costs were as high as € 110.2 billion. This is mainly attributable to the reduction in employment from TRD in the economy. Furthermore, the economic cost of mental illnesses was different from the economic cost of physical chronic illnesses in that the direct cost was higher than the indirect cost [55–58]. This is possibly due to explicit impairment of work due to physical illnesses.

By contrasting the economic costs of TRD and non-TRD among antidepressant-treated MDD, we found TRD imposes significantly higher direct medical costs and indirect costs. This is consistent with existing research [59–61]. Our findings showed that TRD patients utilized more healthcare resource utilization (HCRU) which includes outpatient visits, inpatient hospitalizations, emergency visits, medications, and laboratory tests compared to non-TRD patients. Furthermore, the result confirmed that patients with TRD require more societal support compared to patients with non-TRD.

The novelty of the study's design lies in combining a cross-sectional survey and a retrospective review at the patient-level analysis to estimate the direct and indirect costs of TRD and non-TRD among MDD patients. We estimated the economic costs of MDD, TRD, and non-TRD from microdata representing the actual economic costs of MDD, TRD, and non-TRD. However, the study had several limitations. Firstly, the cost of job loss due to depressive symptoms was excluded, and the indirect cost was calculated using only one unpaid caregiver for a patient with MDD, potentially leading to a probable underestimation of the economic impact of MDD, TRD, and non-TRD and introducing bias in cost estimates. Therefore, including multiple unpaid caregivers and accounting for job loss due to depression symptoms would provide a more accurate economic picture of MDD. Secondly, data on MDD patients were collected solely from a single public tertiary hospital, limiting the generalizability of the findings to other contexts. Moreover, this approach may lead to a potential underestimation of direct medical expenses associated with MDD, TRD, and non-TRD when compared to private hospitals, as public hospitals in Thailand offer more affordable medical services. Thirdly, the tertiary hospital faced a shortage of psychologists, resulting in less psychotherapy activity than was optimal and possibly underestimating direct medical costs. Fourthly, the cross-sectional design lacks established causal relationships between research factors, necessitating the use of prospective or longitudinal designs to demonstrate causality. Fifthly, while the study included antidepressant-treated MDD patients, the sample might not entirely represent the MDD population due to selection bias. Sixthly, the prevalence and economic burden analyses were influenced by medical data from retrospective records, which may have been inconsistent, erroneous, or incomplete. Seventhly, the survey assessing patients' medication adherence was excluded from participant involvement to reduce the potential of recall bias inherent in cross-sectional surveys. This variable has a significant impact on TRD. Incorporating a questionnaire to assess medication adherence is recommended for future studies, as it will considerably improve understanding of treatment resistance in MDD and its impact on patient care. Lastly, the study did not assess the severity of patients' current depression symptoms, or neuro laboratory results, creating several independent variables that TRD could not explain.

To address the study's limitations and advance research in this area, future studies should collect data from multiple healthcare centers to accurately assess the prevalence and economic cost of TRD among MDD patients. This approach will increase the research's scope and enhance its applicability to diverse healthcare settings and individuals. Additionally, a longitudinal or prospective study design can be employed to establish causal relationships between variables and analyze changes over time, providing valuable insights into the development of TRD and its related factors. Lastly, future studies may include evaluations of patients' medication adherence, the severity of current depressive symptoms, and neurobiological test results to gain a deeper understanding of the mechanisms and factors contributing to TRD.

## Conclusions

The present study contributes to understanding the prevalence, clinical characteristics, and economic burden of TRD in a Thai public tertiary hospital. The finding that 19.6% of antidepressant-treated MDD patients met TRD criteria provides valuable insights into this challenging condition's prevalence in this healthcare setting. Characteristic differences between TRD and non-TRD patients revealed significant factors associated with TRD, including age, age of MDD onset, BMI, history of suicide attempts and self-harm, and frequent smoking behavior. These findings can assist clinicians in identifying individuals at higher risk of TRD and guiding personalized treatment approaches. The study also sheds light on the economic burden of TRD, showing that its annualized cost is substantially higher than non-TRD among antidepressant-treated MDD patients. However, the study acknowledges its major limitation in underestimating the economic impact of MDD, TRD, and non-TRD due to the exclusion of job loss costs, the recruitment of only one unpaid caregiver per patient, and survey in a public hospital setting, which may not fully represent the true economic burden.

## Data Availability

The data sets generated during and/or analyzed during the current study are available from the corresponding author.
